# A biochemical framework for eIF4E-dependent mRNA export and nuclear recycling of the export machinery

**DOI:** 10.1261/rna.060137.116

**Published:** 2017-06

**Authors:** Laurent Volpon, Biljana Culjkovic-Kraljacic, Hye Seon Sohn, Alexis Blanchet-Cohen, Michael J. Osborne, Katherine L.B. Borden

**Affiliations:** 1 Institute of Research in Immunology and Cancer (IRIC), Department of Pathology and Cell Biology, Université de Montréal, Pavillon Marcelle-Coutu, Chemin de Polytechnique, Montreal, Québec, H3T 1J4, Canada; 2 Cancer Science Institute of Singapore and Department of Biochemistry, Yong Loo Lin School of Medicine, National University of Singapore, Singapore 117597, Singapore; 3 Department of Human Genetics, Segal Cancer Centre and Lady Davis Institute, Jewish General Hospital, McGill University, Montréal, Québec, H3T 1E2, Canada

**Keywords:** LRPPRC, eIF4E, CRM1, 4ESE RNA, Importin 8

## Abstract

The eukaryotic translation initiation factor eIF4E acts in the nuclear export and translation of a subset of mRNAs. Both of these functions contribute to its oncogenic potential. While the biochemical mechanisms that underlie translation are relatively well understood, the molecular basis for eIF4E's role in mRNA export remains largely unexplored. To date, over 3000 transcripts, many encoding oncoproteins, were identified as potential nuclear eIF4E export targets. These target RNAs typically contain a ∼50-nucleotide eIF4E sensitivity element (4ESE) in the 3′ UTR and a 7-methylguanosine cap on the 5′ end. While eIF4E associates with the cap, an unknown factor recognizes the 4ESE element. We previously identified cofactors that functionally interacted with eIF4E in mammalian cell nuclei including the leucine-rich pentatricopeptide repeat protein LRPPRC and the export receptor CRM1/XPO1. LRPPRC simultaneously interacts with both eIF4E bound to the 5′ mRNA cap and the 4ESE element in the 3′ UTR. In this way, LRPPRC serves as a specificity factor to recruit 4ESE-containing RNAs within the nucleus. Further, we show that CRM1 directly binds LRPPRC likely acting as the export receptor for the LRPPRC-eIF4E–4ESE RNA complex. We also found that Importin 8, the nuclear importer for cap-free eIF4E, imports RNA-free LRPPRC, potentially providing both coordinated nuclear recycling of the export machinery and an important surveillance mechanism to prevent futile export cycles. Our studies provide the first biochemical framework for the eIF4E-dependent mRNA export pathway.

## INTRODUCTION

The eukaryotic translation initiation factor eIF4E plays a major role in post-transcriptional regulation of gene expression (Carroll and Borden 2013). eIF4E is localized in both the nucleus and cytoplasm where it acts in the mRNA export and translation of a specific subset of mRNAs, respectively (Carroll and Borden 2013). Both of these functions require eIF4E to bind the m^7^G cap (cap) on the 5′ end of mRNAs (Culjkovic et al. 2007). eIF4E overexpression alone leads to oncogenic transformation in mouse models and its elevation in cancer is linked to poor prognosis (Carroll and Borden 2013). Thus it is important to understand the mechanisms underlying eIF4E's ultimate effects on the proteome.

Elevated eIF4E-dependent mRNA export contributes to its oncogenic activity. eIF4E is substantially elevated and enriched in the nucleus of some cancer cells, including subtypes of acute myeloid leukemia (AML) and lymphomas (Topisirovic et al. 2003; Culjkovic-Kraljacic et al. 2016). Here, eIF4E-dependent mRNA export is highly elevated and its inhibition in AML patients is correlated with clinical responses (Assouline et al. 2009, 2015). Further, studies with Importin 8, the nuclear importer of eIF4E, revealed that inhibition of the nuclear localization of eIF4E impairs its ability to oncogenically transform cells (Volpon et al. 2016). Mutagenesis studies also support a role for eIF4E mRNA export activity in transformation. For instance, the S53A eIF4E mutant does not act in mRNA export or transform cells; however, this mutant still binds the cap and is active in translation in the cytoplasm (Kaufman et al. 1993; Cohen et al. 2001; Culjkovic-Kraljacic et al. 2012). In the nuclei of mammalian cells, eIF4E specifically associates with over 3000 mRNAs (Culjkovic-Kraljacic et al. 2016). To date, these RNAs contain a 50-nucleotide eIF4E sensitivity element (4ESE) in their 3′ UTR and must be capped. Indeed, eIF4E interacts with its nuclear target transcripts via their cap. For instance, excess m^7^GpppG cap analog competes away 4ESE-containing RNAs immunoprecipitated with eIF4E while GpppG does not (Culjkovic et al. 2005, 2006). Further, the W56A eIF4E mutant that impairs cap-binding does not bind RNAs in either the nucleus or cytoplasm (Culjkovic et al. 2005, 2006). Recognition of selective RNAs underlies a fundamental difference between compartments, i.e., in the nucleus eIF4E only associates with the cap on 4ESE-containing RNAs, while in the cytoplasm it interacts with all capped mRNAs, but only affects the translation of a subset of these (Graff and Zimmer 2003; Culjkovic et al. 2005, 2006).

Despite the clear biological relevance of eIF4E activity, there is no biochemical framework to provide an understanding of the mechanics of this mRNA export pathway. Some important features have been characterized (Culjkovic et al. 2006; Topisirovic et al. 2009) and these serve as a launch point for our studies. First, this activity is independent of ongoing protein synthesis or transcription (Culjkovic et al. 2006). Second, this export pathway does not use the bulk mRNA receptor TAP/NXF1 but is Leptomycin B sensitive, suggesting that the nuclear export receptor CRM1/XPO1 is required (Topisirovic et al. 2009). Third, substrate mRNA requires the cap, as outlined above, and the 4ESE in the 3′ UTR (Culjkovic et al. 2005, 2006). This element is sufficient to promote eIF4E-mediated mRNA export and thus impact on the proteome by increasing the cytoplasmic concentrations of target mRNAs. Fourth, eIF4E only associates with its target mRNAs after splicing, and the complex containing eIF4E and the 4ESE RNA is found in the soluble export competent fraction in the nucleus (Culjkovic et al. 2006; Topisirovic et al. 2009). eIF4E interacts and increases nuclear export of reporter 4ESE constructs that contain no introns, further supporting that ongoing splicing is not a requirement for this pathway (Culjkovic et al. 2006; Topisirovic et al. 2009).

**FIGURE 1.**
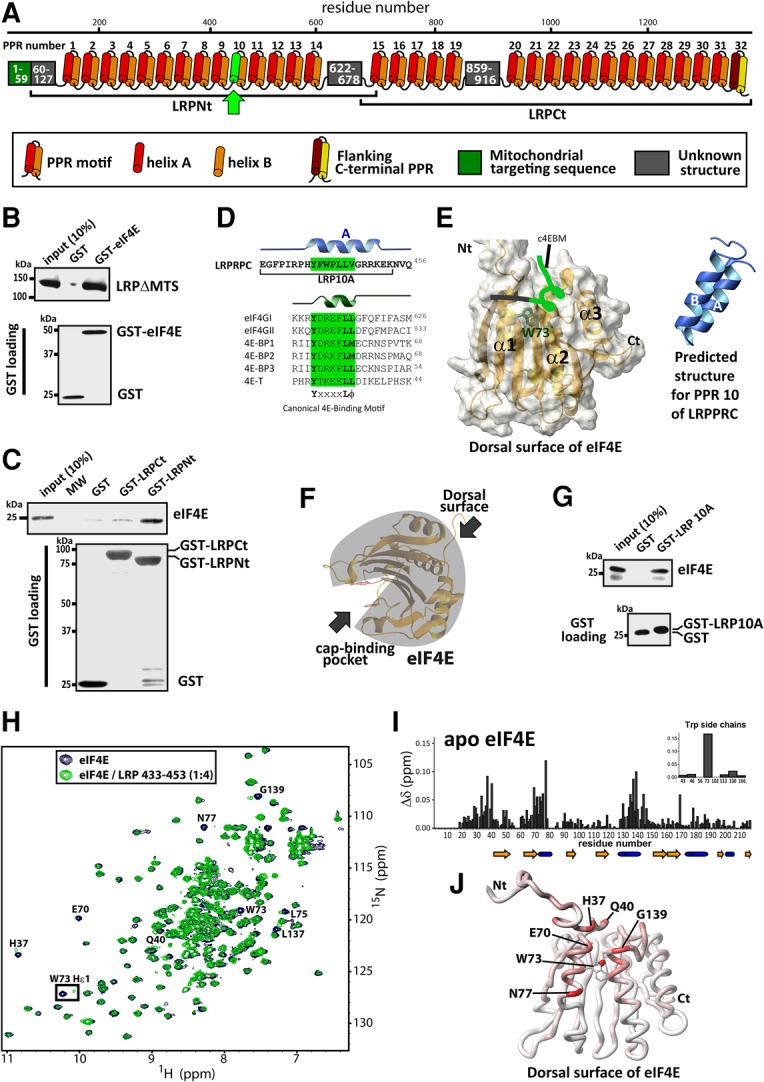
Characterization of the LRPPRC–eIF4E binding site. (*A*) Schematic representation of the human LRPPRC protein. LRPPRC consists of an N-terminal mitochondrial targeting signal (MTS; dark green square), 32 PPR domains represented by red and orange cylinders (helices A and B, respectively), and three domains with unknown structure (gray rectangles). The last PPR is slightly longer (∼42 amino acids) than average PPRs and is thought to cap the structure. The arrow indicates the position of the helix 10A (light green), which contains a putative 4E-binding motif c4EBM (see panel *D*). The two LRPPRC fragments (LRPNt and LRPCt) used are also shown. (*B*,*C*) Association of LRPPRC demonstrated by GST pull-down assay using GST-eIF4E (*B*) or GST-LRPPRC fragments (*C*) as bait. The prey is indicated on the *top* Western blot and the GST loading is shown *below*. LRPΔMTS represents the mature form of LRPPRC. (*D*) The sequence of PPR10 of LRPPRC was aligned on the position of the c4EBM with the eIF4E-interacting regions of eIF4GI-II, 4E-BP1-3, and 4E-T (Gruner et al. 2016). The canonical motif is boxed in green. The secondary structure (predicted for LRPPRC) is shown *above* each sequence, and the fragment LRP10A used in panels *G*–*J* is represented *below*. (*E*) Schematic representation of eIF4E (PDB: 3AM7) bound to 4E-BP2 (*left* side) (Fukuyo et al. 2011). Here, the dorsal surface of eIF4E is shown and all three helices are labeled. The same color-coding is used as in panel *D*. On the *right* side is the predicted structure for PPR 10 of LRPPRC. (*F*) Representation of eIF4E with its cap-binding site being located at the *opposite* side of its dorsal surface where other proteins bind and regulate its activity. (*G*) Association of LRP10A with eIF4E demonstrated by GST pull-down assay. (*H*–*J*) eIF4E backbone amide chemical shift changes during titration with the LRP10A peptide. (*H*) Superposition of eIF4E ^1^H–^15^N HSQC NMR spectra collected for eIF4E/LRP10A ratios of 1:0 (blue) and 1:4 (green). Various cross-peaks affected by complex formation are labeled according to residue number. (*I*) Per-residue plot of chemical shift perturbations at 1:4 ratio. The *inset* indicates the perturbations for the eight Trp side-chains. (*J*) Mapping of the amide chemical shift changes on the backbone of eIF4E (PDB: 2GPQ). The intensity of the red color is proportional to the magnitude of the chemical shift displacement. All experiments were carried out at least three independent times.

To define biochemical factors in this export pathway, we carried out proteomic studies and found that the leucine-rich pentatricopeptide repeat protein (LRPPRC) immunoprecipitates with both eIF4E and 4ESE RNAs in the nucleus (Topisirovic et al. 2009). Importantly, knockdown of LRPPRC reduces the ability of eIF4E to immunoprecipitate with 4ESE RNA and impairs eIF4E-dependent mRNA export (Topisirovic et al. 2009). These studies suggest that LRPPRC is an important factor in this pathway. Indeed, previous studies implicated LRPPRC in mRNA nuclear export as well as in roles in mitochondrial RNA metabolism (Mili and Pinol-Roma 2003; Ochiai et al. 2007; Ruzzenente et al. 2012). The LRPPRC protein is composed almost exclusively of pentatricopeptide repeat (PPR) motifs, except for the flanking N-terminal mitochondrial targeting signal (MTS) and three small regions of unknown function (Fig. 1A; Sterky et al. 2010; Spahr et al. 2016).

In this study, biochemical, biophysical, and NMR methods have been used to characterize the eIF4E export RNP and to determine essential components for RNA recognition and for association with the nuclear pore. We show that LRPPRC is a central component of this RNA export pathway. Specifically, LRPPRC directly binds eIF4E, the 4ESE RNA element, and CRM1. Thus, LRPPRC is the first factor shown to act in 4ESE RNA selection. Further, we reconstituted the eIF4E–LRPPRC–4ESE RNA–CRM1 complex in vitro suggesting that this could be the basic export mRNP in the nucleus. Finally, we show that RNA-free LRPPRC, like cap-free eIF4E, is reimported into the nucleus via Importin 8, suggesting coordinated recycling of these export components. Our studies provide a molecular basis for essential steps in the eIF4E-dependent mRNA export pathway as well as a molecular understanding of how cap- and RNA-binding status underlie critical control points in this process.

## RESULTS

### LRPPRC directly binds eIF4E

We examined whether LRPPRC plays a direct role in eIF4E-dependent mRNA export by testing if it directly interacts with eIF4E in pull-down assays. Recombinant GST-tagged eIF4E and the mature form of LRPPRC (denoted LRPΔMTS due to removal of the mitochondrial targeting signal) were expressed and purified from bacterial cells. LRPΔMTS directly bound eIF4E in this assay (Fig. 1B). Further mapping studies revealed that eIF4E binds the N-terminal region of LRPPRC (LRPNt; residues 67–696) with no binding detected when using GST alone or with the C-terminal fragment LRPCt (residues 673–1394) (Fig. 1C). Inspection of the LRPNt sequence revealed a putative canonical eIF4E-binding motif (c4EBM) starting at Y441 (Fig. 1D,E). These motifs, defined as YxxxxLΦ (where x is any residue and Φ any hydrophobic), are found in many eIF4E binding proteins including eIF4G, and interact with the dorsal surface of eIF4E (Fig. 1F; Carroll and Borden 2013). Given difficulties generating LRPNt suitable for NMR mapping studies, we generated a GST-tagged LRPPRC peptide containing the c4EBM motif, which corresponded to helix 10A of the predicted PPR motif (residues 433–453 underlined in Fig. 1D, denoted as LRP10A; see arrow in Fig. 1A). We observed by pull-down assays that LRP10A was sufficient to bind eIF4E (Fig. 1G). The c4EBM and PPR motifs are mutually exclusive in terms of structures (Fig. 1E), and thus further studies are required to determine the structure of this binding motif.

Next, we determined the binding surface on eIF4E recognized by LRP10A using NMR. Specifically, we observed chemical shift perturbations and line broadening in the HSQC of ^15^N-labeled eIF4E upon addition of the unlabeled LRP10A (Fig. 1H–J). Mapping the chemical shift perturbation onto the eIF4E structure revealed that LRP10A perturbed residues on helix 1, particularly W73, a marker of the dorsal surface, as well as helix 2 and residues 33–40 of the N-terminal arm of eIF4E (Fig. 1I,J). Therefore, our data indicate that LRP10A binds on the dorsal surface of eIF4E, with a footprint that largely overlaps with other eIF4E binding partners including eIF4G, which uses the c4EBM motif and also the viral protein Z, which uses a RING domain (Volpon et al. 2010).

**FIGURE 2.**
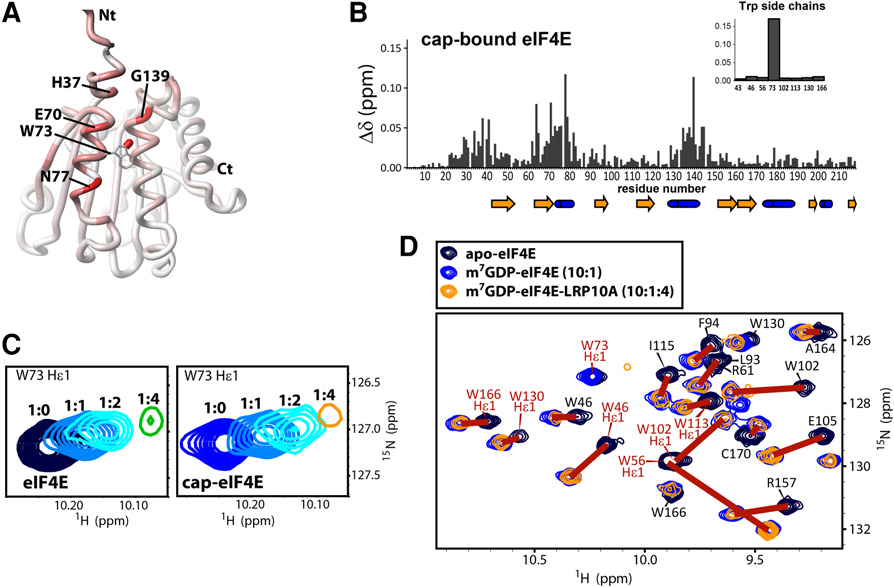
LRPPRC similarly binds capped eIF4E. (*A*) Chemical shift perturbations mapped onto the backbone of the m^7^GDP-bound eIF4E (PDB: 3AM7) upon addition of LRP10A. The same color-coding is used as in Figure 1J. (*B*) Per-residue plot of cross-peak perturbations associated with the titration of m^7^GDP–eIF4E with LRP10A. The *inset* indicates the perturbations for the Trp side-chains. (*C*) To illustrate the similarity in the binding of LRP10A with either cap-free or cap-bound eIF4E, the cross-peak of the side-chain amide proton of W73 is shown at different protein/peptide ratios. (*D*) Representative zoomed region of the HSQC spectra of eIF4E alone (dark blue), bound to m^7^GDP (blue) or in complex with m^7^GDP and LRP10A (orange). Cross-peaks of residues involved in cap binding or close to the cap-binding pocket are labeled, indicating that when bound to LRP10A, eIF4E is also still bound to the cap.

An important feature of eIF4E is the allosteric communication between the cap-binding site and the dorsal surface which are ∼35Å apart (Fig. 1F; Volpon et al. 2006). Indeed, these allosteric changes are responsible for some dorsal surface ligands having differential affinity for cap-free versus cap-bound forms of eIF4E (von der Haar et al. 2004). Thus, we investigated whether LRPPRC also binds cap-bound eIF4E. Using NMR methods, we observed that LRP10A associated with the eIF4E-m^7^GDP cap complex (Fig. 2A–C) using the same surface as it used for cap-free eIF4E (Fig. 1J), including residues W73, H37, Q40, E70, N77, and G139. Importantly, cap-bound and cap-free eIF4E forms are readily distinguishable by HSQC, and we noted that eIF4E was still in the cap-bound form in the presence of LRP10A. Indeed, in the ternary complex m^7^GDP–eIF4E–LRP10A, cross-peaks for residues close to or in contact with m^7^GDP superimposed with those of the m^7^GDP–eIF4E complex (Fig. 2D), indicating that LRP10A did not lead to substantial changes in the cap-binding site of the cap–eIF4E complex. In summary, LRPPRC binds eIF4E either in its cap-free or cap-bound form, through the dorsal surface, a region used by many eIF4E partner proteins.

### LRPPRC directly binds 4ESE RNA via its C-terminal PPR repeats

**FIGURE 3.**
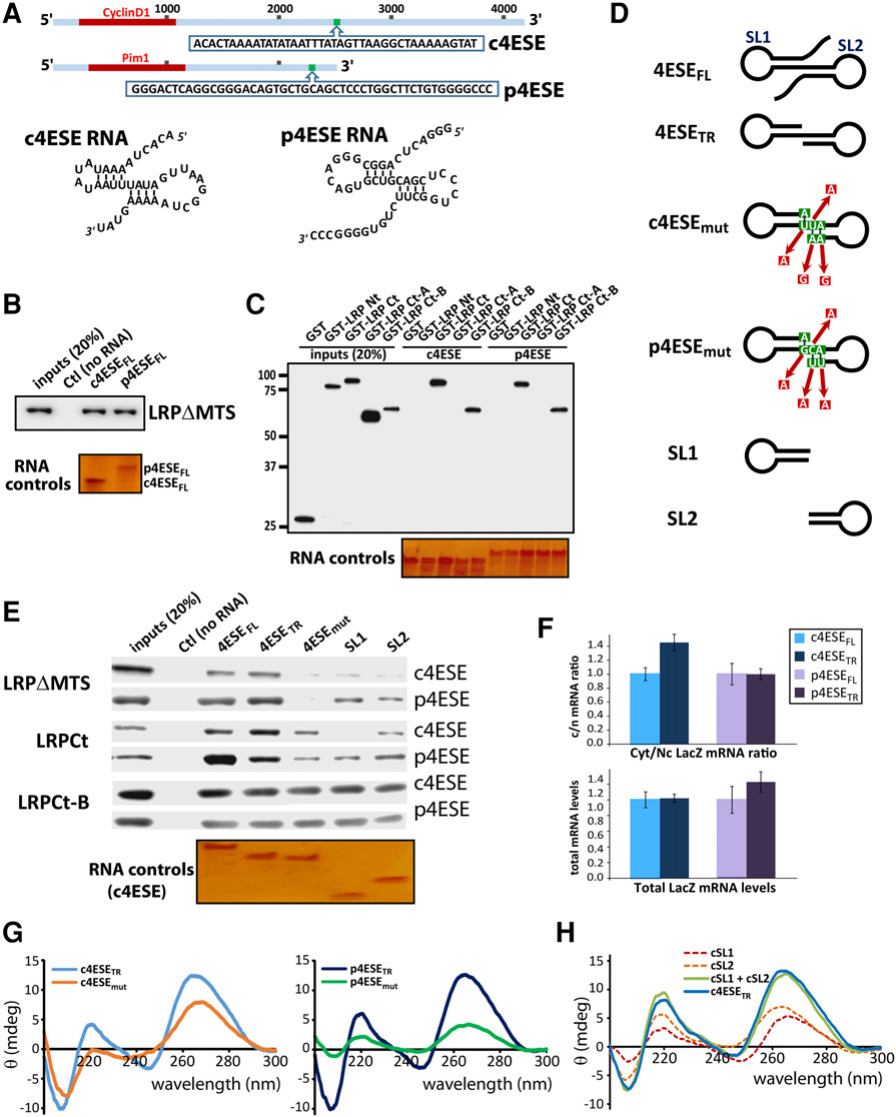
LRPPRC selectively binds to 4ESE RNA. (*A*) Schematic representation of cyclinD1 and Pim1 transcripts. The portion translated is shown in red, and positions of both 4ESEs are in green. Their RNA sequence is shown *below*. The secondary structure for each 4ESE RNA derived from RNase mapping data is shown. (*B*) RNA pull-down between LRPΔMTS and biotinylated 4ESE mRNAs from cyclinD1 and Pim1. RNA controls are shown on denaturing polyacrylamide stained with silver stain (see Materials and Methods for details). (*C*) RNA pull-downs for different GST-tagged LRPPRC fragments and biotinylated c4ESE and p4ESE mRNAs. RNA loading controls as in panel *B*. (*D*) Schematic representation of the different 4ESE RNAs used in this work. (*E*) RNA pull-down using the biotinylated 4ESE mutants described in panel *D* and different LRPPRC fragments. Pull-down samples were analyzed with anti-LRPPRC antibodies (LRPΔMTS) and anti-GST antibodies (LRPCt and LRPCt-B). RNA controls are shown for the above pull-down (LRPΔMTS with c4ESE) and are given as in panel *B*. (*F*) LacZ-4ESE RNA export comparing full-length and truncated forms of the 4ESE elements fused to LacZ in cells overexpressing eIF4E. *Top* panel shows that the mRNA export (cytoplasmic-to-nuclear ratio of RNAs) is not impaired by truncation of the 4ESE element relative to full-length 4ESE. The *bottom* panel shows that total RNA levels are equivalent. RNAs were detected by qPCR. (*G*) Comparison of the circular dichroism spectra of truncated and mutated 4ESE for both cyclinD1 (*left*) and Pim1 (*right*). (*H*) Comparison of the sum of the CD spectra of SL1 and SL2 with 4ESE_TR_.

We next examined whether LRPPRC also specifically binds the 4ESE RNA. In this way, LRPPRC could act as a factor that determines which RNAs associate with nuclear eIF4E. We used 4ESE mRNA export elements derived from cyclin D1 and Pim1 transcripts (c4ESE and p4ESE, respectively) reported previously (Culjkovic et al. 2006). The 4ESE is defined by secondary structure, rather than sequence composition, where both c4ESE and p4ESE form a structurally conserved paired stem–loop based on RNase mapping data (Fig. 3A; Culjkovic et al. 2006). Thus, binding to both 4ESE elements is a strong indication that this common structural motif is the major determinant for recognition. 4ESE elements used in this study were produced synthetically, biotinylated at the 3′ end, and importantly, are not m^7^G capped. First, we established that LRPΔMTS bound to both 4ESEs using biotinylated RNA pull-downs (Fig. 3B). Then, to determine the part of LRPPRC that bound the RNA, we used the LRPNt and LRPCt constructs. In contrast to eIF4E, which binds the N-terminal portion of the LRPPRC, LRPCt construct bound 4ESE RNA (Fig. 3C). Thus, LRPPRC interacts with 4ESE RNAs using PPR motifs 15 to 32 (residues 673–1394).

Next we determined the features of the 4ESE RNA element required for this interaction. First, we investigated the relevance of the 5′ and 3′ regions, which were found to be flexible in the previous RNase mapping studies (Culjkovic et al. 2006). We performed biotinylated RNA pull-downs with full-length (4ESE_FL_) and truncated (4ESE_TR_) forms where the unfolded 5′ and 3′ ends were deleted (Fig. 3D). Here, we observed that LRPΔMTS or LRPCt bound both 4ESE constructs (Fig. 3E). We also showed that both the p4ESE_TR_ and c4ESE_TR_ were at least as active in eIF4E-dependent mRNA export as full-length constructs using standard export assays and qPCR (Fig. 3F). Given that the flexible regions of the 4ESE RNA are dispensable for interaction with LRPPRC and for eIF4E-mediated mRNA export, we used the truncated versions as the basis for generating mutants. For instance, we explored the requirement for the paired-stem–loop structure by mutating nucleotides that would disrupt the base pairs required for folding these elements (4ESE_mut_) (Fig. 3D) based on earlier studies with the Histone H4 4ESE element (Martin et al. 2011). Our CD studies indicate that indeed the 4ESE RNA mutants disrupt the fold (Fig. 3G). Strikingly, c4ESE_mut_ and p4ESE_mut_ associate much less efficiently with either LRPΔMTS or LRPCt compared to 4ESE_FL_ or 4ESE_TR_ constructs (Fig. 3E). Finally, we examined whether either of the two stem–loops alone was sufficient for binding to LRPPRC. We denote these constructs as stem–loop 1 (SL1) or stem–loop 2 (SL2) (Fig. 3D). Interestingly, our CD studies indicated that both SL1 and SL2 were structured and the addition of these spectra led to a spectrum indistinguishable from 4ESE_TR_ (Fig. 3H), indicating that these stem–loops fold independently of each other and retain their folds in the absence of the rest of the 4ESE. However, we observed a weaker association between both LRPΔMTS and LRPCt constructs and either SL1 or SL2, indicating the entire 4ESE_TR_ motif is required.

Next, we attempted to further map the 4ESE RNA binding site within LRPCt. We observed that LRPCt-A (PPR15-22) had no RNA binding activity on its own (Fig. 3C). In contrast, LRPCt-B (PPR23-32) binds all RNAs tested: 4ESE_FL_, 4ESE_TR_, mutant 4ESEs, SL1, and SL2 (Fig. 3C,E). These studies suggest that LRPCt-A confers specificity perhaps by causing alternative folding or exposure of residues in the LRPCt-B fragment when in the larger constructs. Additionally, in the presence of LRPCt-B, LRPCt-A may make contacts with the 4ESE RNA, which convey specificity. Further studies will be needed to understand the structural determinants of LRPCt-4ESE RNA recognition. In summary, we show that LRPCt binds specifically to the folded c4ESE and p4ESE RNAs.

**FIGURE 4.**
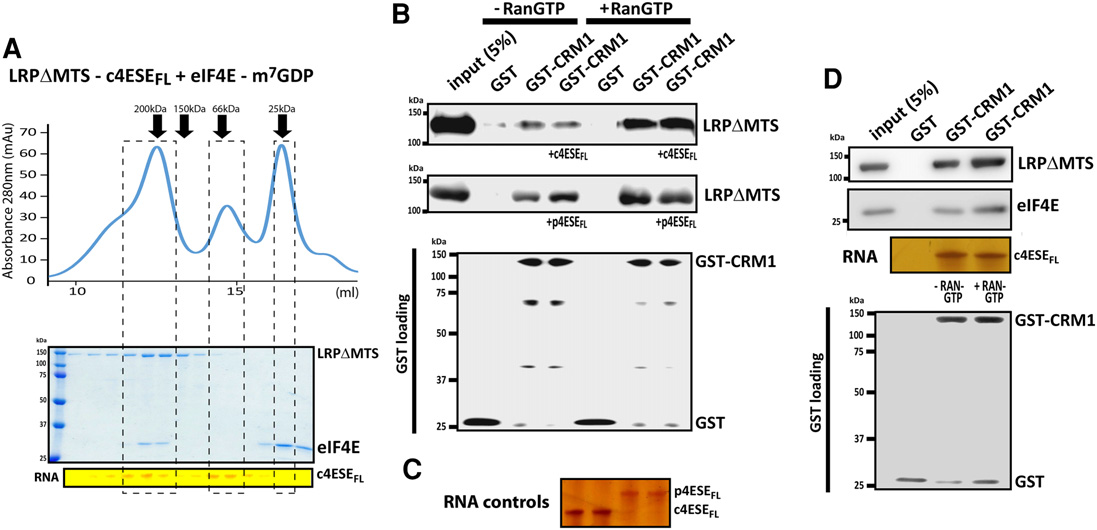
LRPPRC interacts with CRM1 alone and in complex with eIF4E and 4ESE RNA. (*A*) The complex constituted by LRPΔMTS, c4ESE_FL_ RNA, and cap-bound eIF4E was subjected to gel filtration on a Superdex S200 10/300 GL column. The elution profile is depicted (*upper* panel) and protein and RNA contents were analyzed by SDS–PAGE and urea denaturing gel, respectively (*lower* panel). (*B*) GST pull-down assay using GST-CRM1 as a bait and LRPΔMTS as the prey. The effect of adding either the c4ESE_FL_ or p4ESE_FL_ is shown in the *top* and the *middle* blots, respectively, and the 4ESE RNA loading controls (±RanGTP) are shown in panel *C*. (*D*) GST pull-down assay using GST-CRM1 and the LRPΔMTS/c4ESE_FL_/m^7^GDP-eIF4E complex isolated by gel filtration (panel *A*). LRPΔMTS, eIF4E, and CRM1 were observed by Western blot, and RNA as above. Experiments were done at least three independent times.

Finally, we examined whether LRPΔMTS could simultaneously bind eIF4E and 4ESE RNA using gel filtration chromatography (Fig. 4A). After LRPΔMTS and eIF4E were preincubated with excess of c4ESE_FL_ and m^7^GDP cap analog, respectively, both complexes were combined and subjected to gel filtration. We observed a peak corresponding to a size of ∼200 kDa, using globular proteins to calibrate the column. This value is only slightly larger than the expected 158 kDa molecular weight for an equimolar complex of LRPPRC–eIF4E–4ESE RNA. Importantly, the elongated shape that these PPR-containing proteins like LRPPRC typically adopt, likely affect migration on the column. To determine that all the components were indeed present, column fractions were subjected to gel electrophoresis and visualized by Coomassie blue staining to detect LRPΔMTS and eIF4E, and on denaturing polyacrylamide stained with silver stain for the c4ESE_FL_ RNA. All three factors were found in the ∼200 kDa fraction consistent with the formation of the equimolar trimeric complex in solution.

### LRPPRC binds the CRM1 export receptor

Our above studies indicated that LRPPRC binds both eIF4E and the cargo 4ESE RNA. In order to be exported through the nuclear pore, RNA and protein complexes must interact with an export receptor. Given our previous findings that eIF4E-dependent mRNA export is abrogated by inhibition of CRM1 with Leptomycin B (Culjkovic et al. 2006), we examined whether LRPPRC directly binds CRM1 by GST pull-down assays. Here, our studies revealed that LRPPRC directly binds CRM1 in a Ran-dependent manner (Fig. 4B), a property typical of CRM1 cargoes (Fornerod et al. 1997). Next, we examined the effects of both c4ESE and p4ESE addition on the LRPPRC-CRM1 interaction. Consistent with the formation of an active export complex, CRM1 also associated with 4ESE RNA–LRPPRC complexes (Fig. 4B,C). Finally, the complex isolated from the gel filtration column containing the LRPΔMTS, cap-bound eIF4E and the c4ESE RNA (Fig. 4A), was used in a pull-down assay with either GST or GST-CRM1 immobilized on Sepharose beads (Fig. 4D). This complex associated with GST-CRM1 in a Ran-dependent manner (as seen in Fig. 4B), but not with GST. Thus, it indicates that LRPPRC interacts with CRM1 both alone and in complex with eIF4E and 4ESE RNAs, possibly forming a minimum eIF4E-dependent mRNA export complex.

### Cap-free eIF4E binds the 4ESE RNA

**FIGURE 5.**
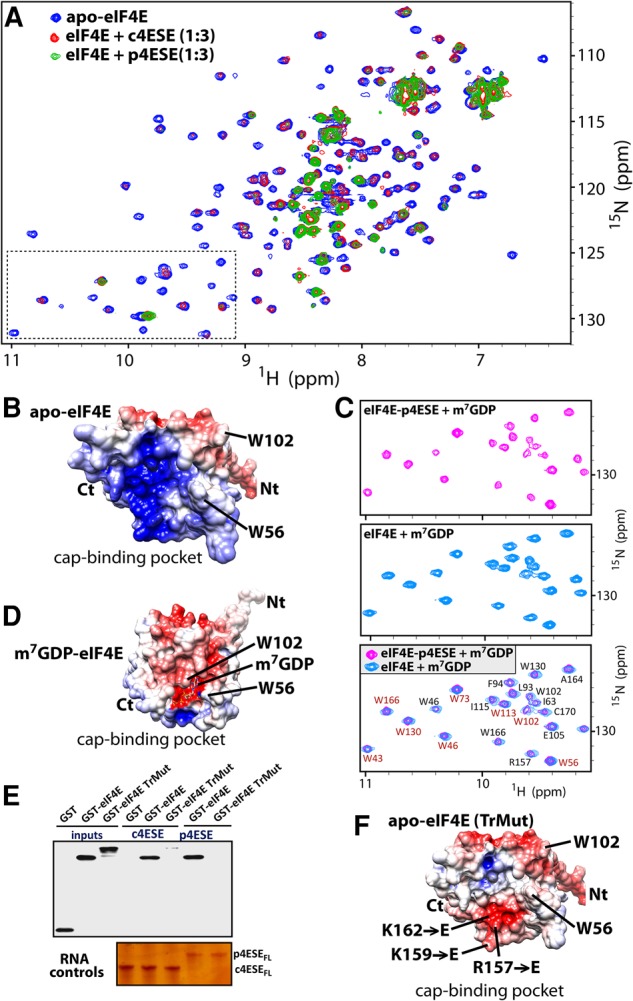
Interaction of eIF4E with 4ESE mRNAs. (*A*) Superposition of the ^1^H-^15^N HSQC spectra of eIF4E in the absence (blue) and presence of 4ESE_FL_ from cyclin D1 (red) or Pim1 (green). (*B*) Surface charge distribution in the cap-binding pocket of apo-eIF4E (PDB: 2GPQ). Key residues for cap binding are shown. Positively and negatively charged regions are in blue and red, respectively. (*C*) Addition of the m^7^GDP to the eIF4E–p4ESE complex (purple) results in an NMR spectra similar to that of the binary m^7^GDP-eIF4E complex (cyan). For clarity, enlarged sections of the HSQC spectra are shown (dashed rectangle in panel *A*). An overlay of both spectra is shown in the *bottom* panel. Assignments are shown for the backbone (black) and Trp indoles (red). (*D*) Surface charge distribution of the m^7^GDP-eIF4E within the cap-binding pocket (as in panel *B*; PDB: 3AM7). (*E*) RNA pull-down between biotinylated 4ESEs and GST-eIF4E wild-type and triple mutant (TrMut). The anti-GST antibody was used, and RNA controls are shown as in Figure 3B. The experiment was done at least three independent times. (*F*) Surface charge distribution of the eIF4E TrMut (same orientation as in panels *B* and *D*). Residues mutated to glutamates are labeled.

All our previous work suggested a model whereby eIF4E associates with the cap of 4ESE containing RNAs in the nucleus. This is based on previous cap competition studies and experiments with eIF4E mutated in the cap-binding site (eIF4E W56A), which indicated that transcripts required capping for eIF4E interactions (Culjkovic et al. 2005, 2006). However, a recent study indicated that eIF4E can also directly bind a 4ESE RNA element located within the coding region of Histone H4 transcripts (Martin et al. 2011). In that case, there is a competition between 4ESE and the cap for eIF4E, where only cap-bound eIF4E could act in translation (Martin et al. 2011). Thus, we examined the possibility that eIF4E also binds the 4ESE RNAs from cyclin D1 and Pim1. Note that the synthetic RNAs used for these studies were not capped. Using NMR, we observed that both c4ESE and p4ESE bound eIF4E (Fig. 5A). Specifically, we observed signal broadening of eIF4E upon binding to 4ESE due to slower tumbling because of the large size of the eIF4E–4ESE RNA complexes (∼37–41 kDa). Intensity of the peaks from the N terminus do not exhibit any broadening, suggesting that the 4ESE is not using this region to interact with eIF4E.

We investigated a role for the cap-binding pocket in eIF4E as a potential 4ESE RNA binding site based on (i) our NMR results and (ii) the observation that this region has a net positive charge (Fig. 5B). First, we added an m^7^G cap analog (m^7^GDP) to the preformed ^15^N-labeled eIF4E-unlabeled 4ESE RNA complex. Cap addition led to dissociation of the eIF4E–4ESE RNA complex as evidenced by the reappearance of resonances but now in cap-bound positions (Fig. 5C,D). Thus, excess m^7^GDP cap disrupted the eIF4E–4ESE RNA interaction, strongly suggesting that the 4ESE RNA interacts with eIF4E near to, or partially overlapping with, the cap-binding site. To verify this, we performed RNA pull-down studies (Fig. 5E) using wild-type eIF4E as well as a triple mutant that alters the charge surface near the cap-binding pocket (Volpon et al. 2016). Specifically, residues R157, K159, and K162, which form a positive surface near the cap-binding site, were all mutated to glutamates to imbue a negative charge on the corresponding surface (Fig. 5F), as it is observed in cap-bound eIF4E (Fig. 5D). Consistent with our NMR results, wild-type eIF4E associates with both c4ESE and p4ESE, while the triple mutant binds neither. Thus, this positive surface on eIF4E is required for 4ESE RNA consistent with the observation that cap and 4ESE RNA binding are mutually exclusive. In summary, our findings support a model whereby if transcripts are properly capped, LRPPRC binds the 4ESE RNA and eIF4E interacts with the m^7^GDP cap. This complex binds CRM1 and export is enabled. However, if RNAs are not capped, there could be a competition with target 4ESE RNAs and eIF4E leading to reduced numbers of LRPPRC–4ESE complexes. Thus, 4ESE binding to eIF4E could provide a surveillance mechanism to ensure that RNA export targets are properly capped.

### Nuclear recycling of LRPPRC is via Importin 8

**FIGURE 6.**
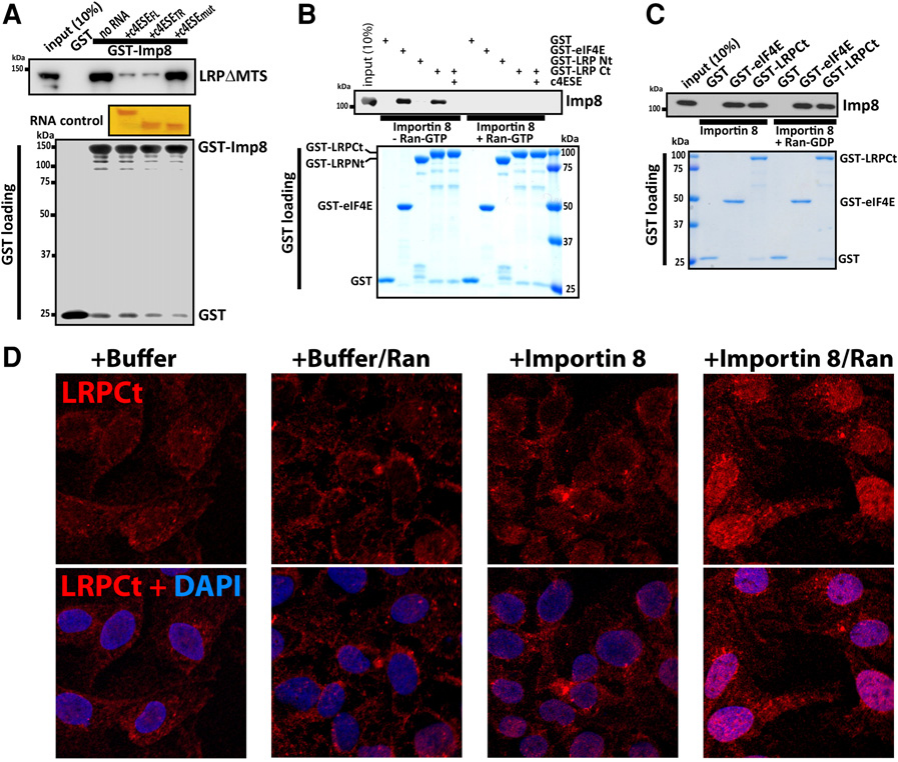
LRPPRC is an import cargo for Importin 8. (*A*) GST pull-down assay for LRPΔMTS and GST-Importin 8, in the absence or presence of wild-type or mutant 4ESE RNAs (see the text for more details). RNA controls were done as in Figure 3B. (*B*) GST pull-down assay for Importin 8 and the two LRPPRC fragments in the absence or presence of c4ESE_FL_ bound to LRPCt. The GST loading and Importin 8 were visualized by Coomassie blue staining, and with the anti-Importin 8 antibody, respectively. (*C*) Same as panel *B* but with RanGDP rather than RanGTP. (*D*) Confocal micrographs monitoring nuclear entry of LRPCt-GST. LRPCt-GST is visualized with a GST antibody in red, and DAPI is the nuclear marker, in blue. Overlay, in pink, indicates that in the presence of Importin 8 and RanGDP, LRPPRC enters the nucleus. Each confocal micrograph is a single section through the plane of the cell with a magnification of 63× and no digital zoom. Experiments were done at least three independent times.

The final step of every export cycle is the reentry of the machinery into the nucleus to support future export rounds. Our previous studies indicated that Importin 8 imports eIF4E (Volpon et al. 2016). Thus, we investigated whether LRPPRC engages the same import pathway and thus if recycling of eIF4E and LRPPRC could be coordinated. We used purified proteins and pull-down strategies with GST–Importin 8 as bait. We observed that LRPΔMTS interacts with Importin 8 (Fig. 6A). Next, we carried out pull-down assays with our LRP fragments as bait. Interestingly, we observed that LRPCt, but not LRPNt, directly binds Importin 8 (Fig. 6B). As expected, we observed that excess Ran-GTP released Importin 8 from its LRPCt cargo, a feature common to importin–cargo interactions (Gorlich et al. 1996). We further showed that Ran-GDP does not release the cargoes, indicating that the effect of Ran-GTP is specific (Fig. 6C). As a positive control, we used eIF4E and showed that it bound Importin 8 in a Ran-GTP dependent manner (Fig. 6B,C). In our previous work, we indicated that association of eIF4E with Importin 8 was driven by electrostatic forces (Volpon et al. 2016). Here, the selectivity of Importin 8 for LRPCt is interesting given both LRPPRC fragments have similar isoelectric points (5.6 and 5.3 for LRPNt and LRPCt, respectively), indicating that charge alone is not sufficient to drive the Importin 8 interaction. Finally, we observed that the addition of excess wild-type c4ESE RNA (fivefold) disrupted the LRPCt–Importin 8 complex (Fig. 6A), suggesting that only RNA-free LRPCt was an Importin 8 cargo. Mutation of c4ESE had no effect on the interaction, consistent with the observation that this RNA mutant does not bind LRPCt (Fig. 3E).

To determine whether these interactions were functional, we carried out in vitro nuclear import assays. Import of LRPCt was monitored in permeabilized U2Os cells using immunofluorescence in conjunction with confocal microscopy. We used GST antibodies to specifically detect exogenous LRPCt. Import assays were performed using a buffer with a mixture of cofactors required for karyopherin-mediated import. In the presence of this buffer, LRPCt remained in the cytoplasm, whereas Importin 8 addition in the presence of Ran led to its nuclear entry (Fig. 6D), consistent with the Ran-dependence requirement for importin cargoes. Thus, LRPCt is an import cargo for Importin 8.

## DISCUSSION

**FIGURE 7.**
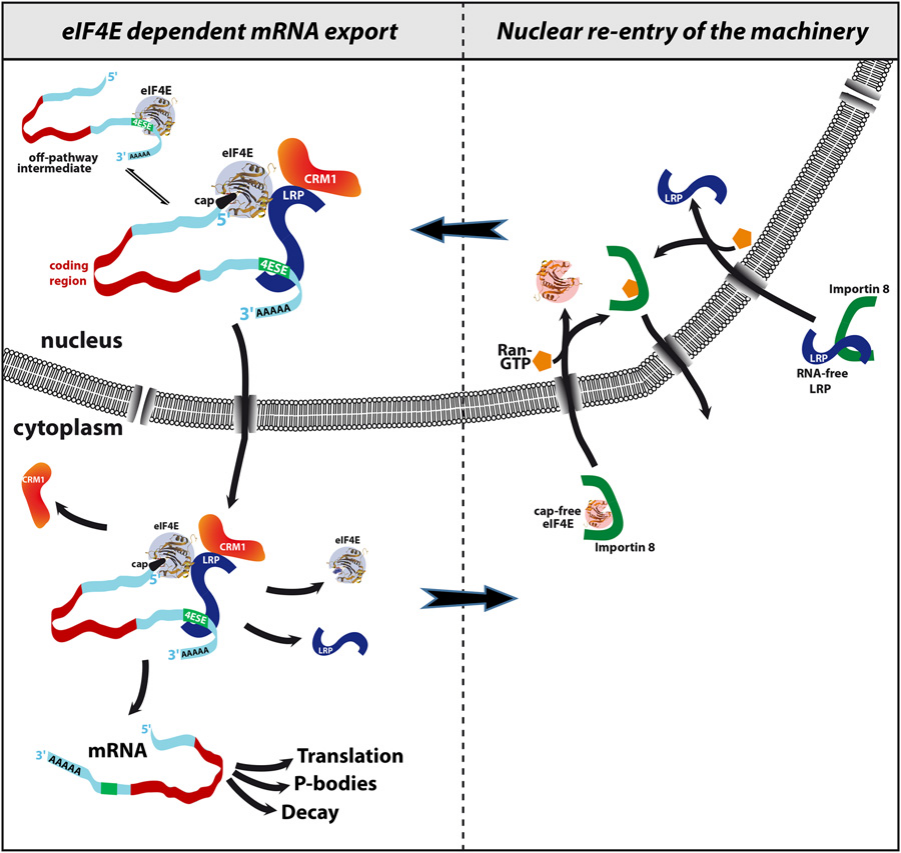
Model for the eIF4E-dependent mRNA export and reentry of the machinery via Importin 8. Apo-eIF4E is in pink and cap-bound eIF4E in purple; the 4ESE element is shown in the 3′UTR as a green box and transcripts are in cyan. This is a minimal model and clearly there could be more cofactors present at each step. We focus on factors and complexes described in the text. The structures for apo and cap-bound eIF4E are from PDB 2GPQ and 3AM7, respectively.

Based on these studies, we propose the first biochemical model for eIF4E-dependent mRNA export. We identify LRPPRC as the RNA specificity component of the complex, directly binding 4ESE RNAs and also as the factor that interacts with the export receptor CRM1. Further, our studies show that LRPPRC binds eIF4E and 4ESE RNA simultaneously through domains in the N and C termini, respectively. LRPPRC also directly binds CRM1, allowing the eIF4E-LRPPRC-4ESE RNA complex to engage the export machinery and transit through the nuclear pore to the cytoplasm (Fig. 7, left side). In this way, LRPPRC acts as a major component of the export machinery, interacting with eIF4E, target RNAs, and CRM1. eIF4E overexpression leads to many alterations to the nuclear pore complex related to CRM1, which likely facilitate the release of RNA cargoes from this receptor once on the cytoplasmic side (Culjkovic-Kraljacic et al. 2012).

Once RNA cargoes are released, the export machinery must be reimported into the nucleus. In our model, after the dissociation of 4ESE RNA cargoes, eIF4E and LRPPRC return to the nucleus via Importin 8 (Fig. 7, right side). Importin 8 only binds cap-free eIF4E (Volpon et al. 2016) and RNA-free LRPPRC (Fig. 6), thereby providing a means to reduce futile export cycles, i.e., reduce reimport of RNA cargoes. Indeed, Importin 8 appears to select positive interaction surfaces (Volpon et al. 2016), and the presence of the RNA substantially impacts the overall charge of the complex, providing a molecular basis for its selectivity. The observation that both LRPPRC and eIF4E use Importin 8 to reenter the nucleus suggests that their import could be coordinated, thereby increasing the efficiency of future export cycles.

An important consideration is how these findings translate from in vitro biochemistry to the cell. Our previous studies in cells support the proposed model in Figure 7. For instance, eIF4E, LRPPRC, and CRM1 immunoprecipitate with each other as well as with 4ESE mRNA in cell nuclei (Topisirovic et al. 2009). These interactions are functionally important for nuclear export. Further, knockdown of LRPPRC reduces the ability of eIF4E to immunoprecipitate with capped 4ESE-containing RNAs in nuclear cell lysates. Conversely, knockdown of eIF4E similarly represses LRPPRC immunoprecipitation with 4ESE RNAs. In both cases, this suppresses eIF4E-mediated mRNA export. Our studies here provide a biochemical explanation whereby eIF4E and LRPPRC are both involved in 4ESE–RNA recognition through binding the cap and 4ESE, respectively. Supporting this model, addition of cap analogs to nuclear lysates suppresses eIF4E's interaction with 4ESE RNAs as does mutation of the 4ESE element (Culjkovic et al. 2005, 2006). Additionally, eIF4E cap-binding mutants do not bind with RNAs in nuclear lysates or retain export activity (Cohen et al. 2001; Kentsis et al. 2001; Culjkovic et al. 2005, 2006). Importantly, eIF4E, LRPPRC, and CRM1 do not immunoprecipitate in the cytoplasm, likely due to rapid disassembly of the export complex. In terms of the other nuclear cap-binding complex CBC, our previous studies indicate that CBC does not immunoprecipitate with eIF4E or LRPPRC and thus CBC does not appear to play a direct role in this process (Topisirovic et al. 2009).

Our model provides many testable features that will be the subject of future investigations. For instance, it would be interesting to isolate eIF4E–LRPPRC–4ESE–CRM1 complexes from cells. In this way, we could determine whether our biochemical models fully recapitulate the in vivo situation. We previously showed that eIF4E's ability to bind 4ESE-containing RNAs is linked to its cap-binding activity (Cohen et al. 2001; Kentsis et al. 2001; Culjkovic et al. 2005, 2006). Thus, it would be important to isolate cap-free eIF4E–4ESE RNA complexes in the cell and establish if they have any functionality beyond a surveillance mechanism. Another consideration for our model is that the specific composition of the eIF4E mRNA export complex may be cell-context dependent. For instance, there could be other 4ESE RNA recognition factors than LRPPRC. These could be relevant for selective export of subgroups of mRNAs or specific cell types. A deeper structural understanding of the LRPPRC-eIF4E interaction would allow identification of sequence or structural determinants that could be found in other potential 4ESE binding proteins. Further, Importin 8 is not expressed in all tissues (Volpon et al. 2016), and thus other Importins presumably substitute for this function as necessary. Thus, it seems likely that there could be context-specific eIF4E-dependent mRNA export scenarios and their investigation will be an exciting future direction. In summary, our studies provide the first biochemical framework for the mRNA export activity of eIF4E.

## MATERIALS AND METHODS

### Recombinant protein expression and purification

Human LRPPRC (residues 60–1394; LRPΔMTS) was expressed and purified as described by Spahr et al. (2016). The different LRPPRC fragments were cloned into the bacterial GST expression vector pGEX-6p-1, and overexpressed in the BL21(DE3) strain of *E. coli*. When bacterial cultures reached OD_600 nm_ of 0.8, expression of recombinant LRPPRC fragments were induced with 0.5 mM isopropyl-β-D-thiogalatopyranoside (IPTG) and allowed to grow at 20°C overnight. The cells were harvested and resuspended in TB-LRP buffer (50 mM Tris pH 7.5, 1 M NaCl, 10% glycerol, 1 mM EGTA, 2 mM DTT) supplemented with protease inhibitors (Roche), DNase, and RNase. The cells were lysed by sonication and supernatant of the lysate added to Glutathione Sepharose 4B (GE Healthcare) for affinity purification. After extensive washing with buffer TB-LRP, followed by WB-LRP buffer (50 mM Tris pH 7.5, 25 mM NaCl, 10% glycerol, 1 mM DTT) to reduce the salt concentration, the GST-tagged LRPPRC fragments were eluted with WB-LRP buffer containing 50 mM reduced glutathione, and loaded onto a Mono Q HP (GE Healthcare) column, followed by gel filtration chromatography (Superdex-200 column; Amersham Biosciences) in 50 mM sodium phosphate pH 7.5, 100 mM NaCl, 10% glycerol, and 1 mM DTT.

Overexpression and purification of the following proteins were as described in Volpon et al. (2016): the ^15^N-labeled human eIF4E (pET-15b) used in the NMR experiments; the mouse GST-tagged eIF4E (pGEX-20T), human GST-CRM1 and GST-Importin 8 (modified pGEX-4T-3 with TEV site), and human Ran (pET-15b) used in the import assays and the different pull-downs. The eIF4E mutants were generated by performing site-directed mutagenesis and verified by DNA sequencing.

### Antibodies and materials

Antibodies used were rabbit polyclonal anti-LRP130 (H-300; Santa Cruz Bio.), rabbit polyclonal anti-Importin 8 (LifeSpan BioSciences), mouse monoclonal anti-eIF4E (BD Transduction Laboratories), goat polyclonal anti-GST (GE Health Sciences), and anti-goat Cy3 (Jackson ImmunoResearch). The cap analog m^7^GDP was obtained from Sigma. The synthetic peptide LRP10A (N-Ac-EGFPIRPHYFWPLLVGRRKEK-NH_2_), in >95% purity was purchased from Biomatic Corporation and stored as a lyophilized powder. The RNA was purchased from Dharmacon, Inc. Prior to use, each nucleotide was heated at 85°C for 5 min, followed by a ramp to 60°C at 0.1°C/sec, 37°C for 5 min, then 25°C for 20 min.

### NMR spectroscopy

^1^H–^15^N HSQCs were recorded at 600 MHz on a Bruker Avance III HD spectrometer equipped with a cryoprobe. Solution conditions were ∼40 µM uniformly ^15^N-labeled eIF4E at 20°C in 50 mM sodium phosphate pH 7.3, 100 mM NaCl, 1 mM DTT, 7% D_2_O. Data were processed using NMRPipe (Delaglio et al. 1995) and analyzed with Sparky (Goddard and Kneller 2003). The graphic representations of the 3D structures were rendered using PyMOL (The PyMOL Molecular Graphics System, Version 1.7.4 Schrödinger, LLC) and Molmol (Koradi et al. 1996). The calculation and analyses of the electrostatic potentials were performed using the programs PDB2PQR (Dolinsky et al. 2004) and APBS (Baker et al. 2001). Chemical shift perturbations for each resonance were calculated using the equation Δδ_obs_ = [(Δδ_HN_^2^ + Δδ_N_^2^/25)/2]^1/2^ (Grzesiek et al. 1996).

### GST pull-down assays

GST and GST-tagged (wild-type and mutant) proteins (75 pmol) were mixed by rotation with its prey (15 pmol) for 1 h at 4°C in 500 μL of binding buffer (10 mM sodium phosphate pH 7.5, 100 mM NaCl, 1 mM EGTA, 1 mM DTT), supplemented with either 0.001% or 0.005% NP40 for the CRM1/LRPPRC or Importin 8/LRPPRC pull-downs, respectively. Then, 20 µL of pre-equilibrated Glutathione Sepharose 4B beads (GE Healthcare) were added and mixed for another 2 h. Unbound protein was removed by washing four times with their corresponding binding buffer and bound proteins were eluted with hot Laemmli sample buffer. The eluted protein complexes were then separated by SDS–PAGE and revealed using specific antibodies and Western blot analysis. In additional competition experiments, Ran-GTP (at fivefold molar excess) was added individually to the binding buffer along with Importin 8 or CRM1 during the incubation step. All experiments were carried out at least three independent times.

### RNA pull-down assays

To remove nonspecific binding, the beads were precleared as follows: 0.01 nmol of protein was mixed by rotation with 20 µL of Streptavidin-agarose beads equilibrated in buffer RNA-X (10 mM sodium phosphate pH 7.5, 100 mM NaCl, 2.5 mM MgCl_2_, 1 mM DTT, 0.05% NP40) for 1 h at 4°C. After the beads were pelleted by centrifugation (500*g*, 3 min), the supernatant was transferred to another Eppendorf tube and 0.1 nmol of biotinylated RNA was added and mixed for another hour. Then, 30 µL of Streptavidin-agarose beads was added, and after incubation for an additional 2 h, beads were washed five times with buffer RNA-X. Pulled-down materials were eluted by boiling with 30 µL Laemmli buffer. Protein content was monitored by Western blot analysis, and RNA loading was visualized on a 20% acrylamide–8 M urea denaturing gel stained with silver nitrate. All experiments were carried out at least three independent times.

### Gel filtration chromatography

Complexes in 500 µL total volume were loaded onto pre-equilibrated (10 mM sodium phosphate pH 7.5, 100 mM NaCl, 1 mM DTT) Superdex 200 10/300 GL column (Amersham Biosciences). The separation was performed at a flow rate of 0.25 mL/min. The amounts of LRPPRC and eIF4E were 0.4 and 1.2 nmol, respectively. The c4ESE_FL_ RNA and the m^7^GDP cap analog were in 3× and 10× excess compared to LRPPRC and eIF4E, respectively.

### Circular dichroism

CD spectra were recorded on a Jasco-810 spectropolarimeter using a 0.1 cm path length quartz cuvette (Hellma). The temperature was maintained at 20°C with a Peltier thermostated cell holder. The oligonucleotides were dissolved in 10 mM sodium phosphate, 50 mM NaCl, 2.5 mM MgCl_2_, to achieve 20 µM sample concentration. Measurements were taken with a 1-nm data interval, and spectra, established as an average of three CD scans, were subtracted with the spectrum of the buffer. All experiments were carried out at least three independent times and representative data are shown.

### Nuclear import assays

U2Os cells were grown on coverslips to 80%–90% confluence. Cells were washed two times in PBS and one time in Transport buffer (20 mM HEPES pH 7.3, 110 mM potassium acetate, 2 mM magnesium acetate, 1 mM EGTA, 2 mM DTT, and a homemade protease inhibitor cocktail containing the following compounds: PMSF, pepstatin A, aprotinin, leupeptin, phenanthroline, and benzamidine) and permeabilized with digitonin (50 µg/mL) for 5 min at RT. The permeabilized cells were washed six times with ice cold Transport buffer. The cells were incubated with import mix at 37°C for 20 min in a humid chamber. Import mixtures contained an energy-regenerating system consisting of the following components: 1 mM ATP, 0.2 mM GDP, 0.2 mM GTP, 5 µM creatine phosphate, and 20 U/mL creatine-phospho kinase (Sigma). The final concentrations for each protein in the mix were 1.5 µM GST-LRPCt, 1.5 µM Importin 8, and 2 µM Ran. After washing three times with Transport buffer, cells were fixed with 4% paraformaldehyde 10 min at RT, then washed three times with PBS to remove paraformaldyde and permeabilized with 0.2% (v/v) Triton X-100 for 10 min at RT. Upon permeabilization, cells were washed four times with PBS, blocked with Blocking buffer (10% FBS and 0.1% Tween 20 in PBS) for 1 h, and incubated with anti-GST antibody (1:1000 dilution) overnight at 4°C in wet chamber, followed by three washes in PBS. Cells were then incubated with Cy3 conjugated anti-goat antibody (1:200 dilution) for 1 h at RT. The cells were then washed four times with 1×PBS and mounted in mounting media with DAPI (Vector Laboratories). Analysis was carried out using a laser-scanning confocal microscope (LSM700; Carl Zeiss) exciting 405 and 543 nm with a 63× oil objective and 1× digital zoom. Channels were detected separately, with no crosstalk observed. Confocal micrographs represent single sections through the plane of the cell. All experiments were carried out three independent times and representative data are shown.

### RNA export assays

The 4ESE-LacZ RNA export assays were followed as described in Culjkovic et al. (2006). All experiments were carried out three independent times.
